# Bronchiolitis: A Real-Life Report of Increasing Compliance to Treatment Guidelines

**DOI:** 10.3390/children12050571

**Published:** 2025-04-28

**Authors:** Melodie O. Aricò, Francesco Accomando, Daniela Trotta, Anthea Mariani, Claudia Rossini, Claudio Cafagno, Letizia Lorusso, Enrico Valletta, Desiree Caselli, Maurizio Aricò

**Affiliations:** 1 Department of Pediatrics, G. B. Morgagni—L. Pierantoni Hospital, AUSL Romagna, 47121 Forlì, Italy; francesco.accomando@auslromagna.it (F.A.); enrico.valletta@auslromagna.it (E.V.); 2 School of Pediatrics, University of Bologna, 40138 Bologna, Italy; 3 Pediatrics, S. Spirito Hospital, Azienda Sanitaria Locale Pescara, 65124 Pescara, Italy; daniela.trotta@asl.pe.it (D.T.); anthea.mariani@asl.pe.it (A.M.); maurizio.arico@asl.pe.it (M.A.); 4 Pediatric Infectious Diseases, Children’s Hospital Giovanni XXIII, Azienda Ospedaliero Universitaria Consorziale Policlinico di Bari, 701243 Bari, Italy; c.rossini4@studenti.uniba.it (C.R.); claudio.cafagno@policlinico.ba.it (C.C.); desiree.caselli@policlinico.ba.it (D.C.); 5 School of Medical Statistics and Biometry, Interdisciplinary Department of Medicine, University of Bari Aldo Moro, 70124 Bari, Italy; l.lorusso80@studenti.uniba.it

**Keywords:** bronchiolitis, guideline application, HFNC, steroids, antibiotics

## Abstract

Background: Bronchiolitis accounts for a substantial number of pediatric hospitalizations and its epidemiology closely parallels that of respiratory syncytial virus (RSV), its principal etiological agent. International guidelines recommend supportive therapy based primarily on oxygen supplementation and hydration. Methods: This study aimed to assess, across three pediatric wards, the impact of internal monitoring and targeted educational interventions on adherence to bronchiolitis management guidelines. Focus was placed on evaluating the effectiveness of tailored strategies in enhancing the appropriateness of treatment practices. Each center independently developed an audit and feedback strategy aligned with local practices and available resources. In Center 1, monthly staff meetings included guideline refreshers throughout the epidemic season. Center 2 appointed two attending physicians to monitor treatment prescriptions and report deviations. Center 3 established an internal protocol through staff consensus, followed by monthly review sessions. In this retrospective analysis, all consecutive patients admitted with bronchiolitis during the winter seasons of 2022–2023 and 2023–2024 (Period 2) were compared with those admitted in 2021–2022 (Period 1). Results: A total of 623 infants under 24 months of age were included, 451 (72%) of whom tested positive for RSV. Their median length of hospital stay was 6 days; 26 infants (4%) required intensive care, and no deaths were recorded. A comparative analysis of the treatment modalities used—high-flow nasal cannula (HFNC) oxygen therapy, inhaled medications, corticosteroids, and antibiotics—revealed a reduced use of non-recommended therapies (e.g., nebulized β2-agonists, steroids, and antibiotics) in Period 2, alongside heterogeneous patterns in HFNC use. Center-specific strategies, tailored to team dynamics and resource availability, effectively promoted greater adherence to evidence-based guidelines. Conclusions: Our findings suggest that structured internal interventions can lead to more appropriate bronchiolitis management and the improved standardization of care.

## 1. Introduction

Bronchiolitis is a clinical syndrome of respiratory distress that predominantly affects young children. Although some discrepancies exist regarding age definitions (e.g., <12 months vs. <24 months), the consensus is to define it as affecting children under 2 years of age [1,2].

The condition typically begins with upper respiratory symptoms, such as rhinitis, which are soon followed by signs of lower respiratory tract infection and inflammation. Bronchiolar involvement results in wheezing and/or rales. Respiratory syncytial virus (RSV) is the most common cause of bronchiolitis, although other pathogens like *Mycoplasma pneumoniae* can also contribute to the syndrome.

In otherwise healthy infants, bronchiolitis is generally a self-limiting illness that resolves without complications. Most non-severe cases can be managed at home with supportive care, including adequate hydration, nasal congestion relief, and close monitoring of disease progression. However, bronchiolitis remains a leading cause of hospitalization in children under 2 years of age [3]. Infants born prematurely or those with underlying cardiopulmonary diseases or immunodeficiencies are at increased risk of complications, including respiratory failure and secondary bacterial infections. Approximately 30% of previously healthy infants hospitalized with bronchiolitis are at risk of developing recurrent wheezing later in life [4]. Despite the disease’s burden, mortality rates for children hospitalized with RSV bronchiolitis in high-income countries are low, typically under 1 per 1000 cases [5,6].

Current guidelines recommend supportive care as the primary management approach to bronchiolitis, including nasal suctioning, nutritional support, adequate hydration, and supplemental oxygen as needed [7,8,9]. In severe cases, hospitalization for enhanced monitoring and support is required [7]. Evidence-based recommendations do not support the routine use of inhaled bronchodilators, nebulized adrenaline, corticosteroids (inhaled or systemic), or antibiotics [7,8,9]. However, numerous studies report the continued use of non-recommended treatments in different countries [10,11,12,13,14].

In a previous study, we assessed real-world therapeutic practices in a network of Italian pediatric wards [14]. The goal of the present study is to evaluate, within three of these wards, the impact of internal monitoring and targeted interventions aimed at improving adherence to clinical guidelines. Specifically, we aim to examine the effectiveness of these reinforced practices and their influence on clinical behavior.

## 2. Materials and Methods

### 2.1. Aim and Study Design

This retrospective study aimed to assess the impact of internal monitoring and local efforts on improving adherence to established bronchiolitis treatment guidelines across three pediatric wards. The study specifically focused on evaluating the effectiveness of reinforced practices and their influence on guideline compliance.

### 2.2. Setting

Data were collected from three pediatric wards in Italy:

- Center 1 (Bari): Pediatric Infectious Diseases Unit within a Children’s Hospital, part of a tertiary-level teaching hospital.
- Center 2 (Forlì): Pediatric inpatient ward within a second-level general hospital.
- Center 3 (Pescara): Pediatric inpatient ward within a second-level general hospital.

### 2.3. Study Population

All consecutive children diagnosed with bronchiolitis and admitted to the participating centers between October 2021 and February 2022 were included in the Period 1 group [14]. All those admitted between March 2022 and April 2024 were included in the Period 2 group.

### 2.4. Participants

The inclusion criteria were an age of less than 2 years; clinical diagnosis of bronchiolitis according to current guidelines [7,8,9]; hospital stay ending in either routine discharge, referral to the ICU, or death; and parental consent for the use of clinical data for research purposes.

The exclusion criteria were respiratory failure due to pre-existing pulmonary, cardiac, or neuromuscular conditions; refusal of treatment; and voluntary discharge before completing treatment.

Data Collected. The following variables were collected: Demographics—age, sex, weight; neonatal risk factors—prematurity, cardiopulmonary comorbidities; diagnostics—RSV status, detection of other respiratory pathogens; treatment details—oxygen therapy, inhaled β2-agonists, systemic antibiotics or steroids, ventilation mode, ICU admission, and length of hospital stay.

Laboratory Testing. RSV and other viral respiratory pathogens were identified using nasal swabs or aspirated secretions, which were analyzed by multiplex PCR using commercially available diagnostic kits.

Treatment Protocol. While treatment protocols were not standardized across centers, all three centers aimed to follow current evidence-based guidelines [7,8,9]. The hospitalization criteria used included signs of respiratory distress, inadequate oxygen saturation, feeding difficulties, and the presence of comorbidities (e.g., neurological, cardiac, or pulmonary conditions).

International guidelines classify the use of steroids, antibiotics, and inhaled bronchodilators as non-evidence-based treatments for bronchiolitis. However, these treatments are not categorically excluded in certain clinical scenarios, such as bacterial co-infections or recurrent wheezing. These cases are expected to be rare, and the proportion of patients receiving these treatments was analyzed. The criteria for ICU admission were not strictly standardized.

Strategies to Increase Guideline Adherence. Each center independently developed and implemented the following strategies to improve their adherence to international bronchiolitis treatment recommendations: Center 1— review sessions of guideline content were held at the start and throughout the epidemic season during staff meetings. Center 2—two attending physicians were tasked with monitoring treatment prescriptions and providing regular feedback to medical staff. Center 3—a local protocol was created through staff consensus, with monthly discussions to reinforce its application.

Statistical Analysis. This study was conducted in accordance with the guidelines of the Declaration of Helsinki. Patient consent was waived as the data were anonymized.

Data were analyzed by period and by hospital. Categorical variables were presented by period using frequencies and percentages, with comparisons between independent samples performed using the chi-square test or Fisher’s exact test as appropriate. The Kolmogorov–Smirnov test was used to check for normality in quantitative variables. If normality was assumed, the mean and standard deviation (SD) were used; otherwise, the median and interquartile range (IQR) were displayed. Parametric methods, such as the t-test, were used for normally distributed variables, while non-parametric tests, such as the Wilcoxon test with continuity correction, were applied where normality was not assumed. The *p*-value threshold for statistical significance was set at <0.05. All analyses were conducted using SAS^®^ software version 9.4, and graphs were created using R Software version 4.4.1 [15].

## 3. Results

A total of 623 children admitted to the three participating centers with bronchiolitis were included in this study: Center 1 (Bari), *n* = 265; Center 2 (Forlì), *n* = 163; Center 3 (Pescara), *n* = 195. Table 1 provides a detailed overview of the demographic and clinical characteristics of the study population across the three centers and two study periods, highlighting significant differences in age distribution, weight, and the prevalence of neonatal risk factors. Seventy-two children (11%) had underlying conditions that may have predisposed them to or complicated the course of their bronchiolitis; the types of risk factors seen are listed in Table 1. RSV was detected in 72% of the total cohort. This proportion was slightly higher in Period 1 compared to Period 2 (overall *p* = 0.0007). However, in all subgroups, RSV positivity remained above 62%.

A thorough investigation of respiratory pathogens other than RSV was not performed in Center 3 during Period 1. Center 1 screened all patients with bronchiolitis for a panel of respiratory pathogens, while Center 2 performed this investigation mainly, but not only, in patients testing negative for RSV. Center 3 began testing for other pathogens at the beginning of Period 2. Other viral pathogens were thus identified in a total of 140 patients (22%). Their distribution is summarized in Table 2.

To evaluate diagnostic practices, particularly the use of routine laboratory testing, we assessed the frequency of C-reactive protein (CRP) measurements. Among the 428 patients for whom this information was available, CRP levels were reported in 235 cases (55%).

The main components of the treatment administered to children with bronchiolitis are summarized in Table 3 and stratified by study period.

The use of antibiotic therapy showed a marked decrease over time, falling from 50% in Period 1 to 24% in Period 2 (*p* < 0.0001). Similarly, the use of systemic steroid therapy significantly decreased from 32% to 19% (*p* = 0.0005).

A substantial reduction was also observed in the use of inhaled therapies, which were administered to 69.8% of patients in Period 1 compared to 16.6% in Period 2 (*p* < 0.0001). Importantly, these reductions in non-evidence-based treatments did not affect the rate of ICU admissions, which remained stable, at 4%, across both study periods.

The use of various therapeutic components was also explored across the three participating centers (Table 4).

Antibiotic Therapy: in Center 2, the use of antibiotics remained stable, from 27% to 21%, while a significant reduction was observed in the other two centers—from 46.7% to 27% in Center 1 and from 66% to 22% in Center 3.

The use of systemic steroids remained stable in Centers 1 and 3, albeit at different rates (around 17% in Center 1 and around 30% in Center 3). However, in Center 2, steroid use dropped dramatically, from 50% to 11.7% (*p* < 0.0001).

Data on inhaled therapy with bronchodilators were available in full for two centers. In both centers, the prescription rate for this treatment dropped significantly from approximately 70% to between 12% and 25%.

The need for ICU admission decreased significantly in Center 1 (from 6% to 1%; *p* = 0.05), remained very low in Center 2 (from 0% to 2.5%), and showed a non-significant increase in Center 3 (from 5.6% to 9.7%; *p* = n.s.).

The median duration of hospital stays varied across centers. Center 1 reported stays of between 6 and 7 days, Center 2 maintained its remarkably short stays (3 days), and Center 3 reduced its length of stay significantly, from 8 to 6 days.

In total, 26 children (4%) required ICU admission, and no deaths were reported. An interesting trend was the significant decrease in the proportion of patients tested for C-reactive protein (CRP) in one center, which fell from 100% to 35% over the observation period.

## 4. Discussion

Bronchiolitis continues to present significant challenges for pediatric wards and the families of affected children, given its high incidence and impact on healthcare resources. Our cooperative group of these three pediatric wards, scattered over the country, has been working in this field, trying to increase the awareness of current guidelines and to achieve a reduction in unnecessary therapies for bronchiolitis [14].

In this study, we evaluate the data of 623 children hospitalized for bronchiolitis during the winter season in 2021–2022 (serving as control group) versus the winter seasons in 2022–2023 and 2023–2024. As expected, the overall median age of the affected children was 3 months and about three-quarters of the children tested positive for RSV, with no significant difference between the three centers and the three epidemic waves. Likely due to the increasing level of neonatal care provided to infants, the proportion of children with individual risk factors which may predispose them to, or complicate, bronchiolitis was not negligible, accounting for 11% of cases. This may have implications in terms of the risk of a complicated course of the disease. Nevertheless, only 26/623 (4%) children were admitted to an ICU.

In the diagnostic approach to bronchiolitis, biochemistry and laboratory tests, beyond exploring whether it is RSV or other pathogenic infection, are usually not required. In our patients, 55% of children had their CRP assessed. Interestingly, the proportion of patients tested for CRP varied widely, even within the same center, suggesting a gradual shift in diagnostic practices. This variation may reflect their evolving adherence to clinical guidelines, which emphasize that CRP tests often lack predictive value for the severity of bronchiolitis. Similar findings have been noted in other studies, where CRP was found to have limited utility as a marker of disease progression in viral infections like bronchiolitis. This emphasizes that CRP’s limited role aligns with current guidelines and other studies. In another recent study, we reported that the use of CRP as a supposed marker of disease aggressiveness was not predictive of the clinical course and thus not useful, or even potentially misleading, in children with adenovirus infection, another common epidemic infectious disease in small children [16]. A recent study by Adar et al. on 1874 children hospitalized with RSV bronchiolitis demonstrated that CRP levels did correlate with some adverse outcomes, such as longer hospitalization and ICU admission, but these findings are not universally accepted as sufficient enough to change treatment paradigms [17].

Oxygen therapy remains a cornerstone of the treatment for children requiring hospitalization due to bronchiolitis. The introduction of HFNC therapy aimed to reduce ICU admissions. In our experience, its use was increased overall, though not significantly, in the second period compared to the first control period. Its use, however, was not uniform across centers and varied widely (18% to 88%) between centers during the second period, suggesting that the availability of HFNC machines, rather than disease severity, influenced its adoption. A recent Cochrane review demonstrated that HFNC therapy results in a modest reduction in hospital stay compared to low-flow nasal cannula oxygen and reduces the need for treatment escalation [18].

A major focus of our study was the reduction in the use of non-evidence-based therapies in non-complicated bronchiolitis, such as systemic steroids and antibiotics. Despite long-standing evidence against the use of these therapies in bronchiolitis, many pediatricians continue to prescribe them, often due to insufficient knowledge of guidelines or defensive practices [19,20,21,22,23,24]. In our study population, systemic steroids were given to approximately 21.9% of patients. Importantly, the proportion of patients receiving steroids decreased significantly over the study periods, from about one-third to 19%. This reduction can be seen as a major achievement, reflecting growing awareness about the limited efficacy of systemic steroids in bronchiolitis and aligning with updated guidelines.

Antibiotic use, which is often prompted by respiratory distress and fever—symptoms that may suggest a bacterial co-infection—was also a concern. About 50% of children received antibiotics during the 2021–2022 period. However, by the 2022–2023 period, this proportion decreased significantly, to 24%. This significant reduction (*p* < 0.0001) was more pronounced in two centers, as antibiotic use dropped from 47% to 27% in Center 1 (*p* = 0.003) and from 66% to 22% in Center 3 (*p* < 0.0001). These findings support the concept of antibiotic stewardship and highlight the benefits of continuous education and monitoring in reducing unnecessary antibiotic prescriptions [25].

The use of inhaled therapies, particularly bronchodilators, remains a debated topic in bronchiolitis management [26,27]. In our centers, the use of inhaled therapies also decreased significantly (*p* < 0.0001) in the second study period compared to the first. This change aligns with current guidelines recommending against the routine use of inhaled bronchodilators in bronchiolitis, as evidence suggests that these treatments provide little benefit in most cases.

The overall ICU admission rate remained low but varied across centers, ranging from 1.5% to 9.8% in the second period. This variability may reflect differences in the centers’ attitudes toward the use of CPAP on the ward, as well as logistical factors related to the location of the ICU (whether it is in the same building or requires a transfer). Despite this variability, the overall ICU admission rate remained low, suggesting that the improvements in treatment strategies were effective.

The reduction in hospital stay duration across centers highlights the benefits of evidence-based treatment protocols not only in improving patient management but also in reducing the burden on healthcare resources, especially during peak epidemic seasons.

Beyond efficacy, we aimed to achieve a more appropriate therapeutic approach. Treatment improvement was demonstrated by a lower proportion of children receiving unnecessary drugs such as antibiotics and steroids. The selected approach to improving compliance to guidelines was different in the different centers [28]. After having reviewed, in a previous study [14], the attitudes of our three centers towards treating bronchiolitis, we agreed to define interventions as potentially more effective and applicable based on the analysis of locally available resources. In Center 1, refreshing staff’s knowledge of the guidelines resulted in a significant reduction in the prescription of antibiotics, steroids, and inhaled therapy; in Center 2, assigning two attending physician to survey prescriptions and regularly report to the staff resulted in a significant reduction in all “non-necessary prescriptions”; in Center 3, defining a domestic protocol resulted in reduced prescriptions of antibiotics, but not reduced prescriptions of steroids to the same degree, while data on the use of inhaled therapy could not be quantified.

This study has some limitations: The multicenter setting gave us the opportunity to gather for a large study population but did result in different approaches to self-monitoring. Furthermore, although the three teams agreed on general approaches to bronchiolitis during children’s hospitalization, some decision-making steps (e.g., the criteria for choosing respiratory support or resorting to intensive care) required a reasonable degree of decision-making freedom to be granted to attending physician according to each patient’s specific clinical situation.

## 5. Conclusions

This study provides valuable insights into the effectiveness of internal monitoring and guideline adherence in improving bronchiolitis management. The reduction in unnecessary treatments, such as antibiotics and steroids, and the improvement in treatment consistency reflect significant progress toward more appropriate therapeutic approaches. The strategies used to improve adherence to guidelines varied across the three centers, with each center implementing tailored interventions based on their local resources. The reduction in hospital stays and the low ICU admission rates recorded further support the success of these initiatives. Moving forward, we plan to continue monitoring and refining our approach, particularly in light of newer interventions like RSV immunization, to further enhance the care of children with bronchiolitis.

## Figures and Tables

**Table 1:** Demographic and clinical characteristics of 623 children with bronchiolitis admitted to three Italian pediatric wards during Period 1 (2021–2022) and Period 2 (2022–2024).

Variable	Center									Total
	1 (*n* = 265)			2 (*n* = 163)			3 (*n* = 195)			623
	Period 1 (n = 62)	Period 2 (n = 203)	*p* Value	Period 1 (n = 44)	Period 2 (n = 119)	*p* Value	Period 1 (n = 71)	Period 2 (n = 124)	*p* Value	
Age in months, median (IQR)	2 (1–4)	3 (2–6)	**<0.0001**	5 (2–11.5)	3 (2–7)	0.14	3 (1–5)	3 (2–7)	**0.02**	**3 (2**–**6)**
Male gender, n (%)	32 (53)	113 (56)	0.58	29 (66)	72 (71)	0.53	39 (55)	74 (60)	0.52	359 (58)
Weight in kg, median (IQR)	4.5 (3.4–9)	6 (5–7.8)	0.29	7.5 (5.5–11)	6.5 (5–8)	**0.01**	5.8 (4.7–8.1)	6.2 (5–7.7)	0.38	6.1 (5–8)
Neonatal risk factors, n (%)	3 (5)	26 (13)	0.1 *	3 (7)	16 (13)	0.29	4 (5.6)	20 (17)	**0.04**	**72 (11)**
Type of risk factor, n										
• cardiac		1			3		2	7		
• neurologic		1					1	4		
• respiratory		1					0	0		
• preterm	2	20		2	9		1	3		
• other	1	3		1	4		0	6		
RSV-positive, n (%)	54 (87)	128 (62)	**<0.001**	35 (79)	95 (79)	0.97	53 (80)	86 (69)	0.10	448 (72)

Legend—RSV: respiratory syncytial virus; IQR: interquartile range. Bold *p*-values indicate statistically significant differences (*p* < 0.05). * The difference has been calculated with Fisher’s Exact Test.

**Table 2:** Detail of pathogens other than RSV identified in 623 children with bronchiolitis admitted to three Italian pediatric wards during Period 1 (2021–2022) and Period 2 (2022–2024), organized by RSV status.

	Center 1				Center 2				Center 3			
Pathogen	Period 1		Period 2		Period 1		Period 2		Period 1 *		Period 2	
	*RSV* +	*RSV* −	*RSV* +	*RSV* −	*RSV* +	*RSV* −	*RSV* +	*RSV* −	*RSV* +	*RSV* −	*RSV* +	*RSV* −
Adenovirus	1	0	1	1	1	0	1	0			0	0
Influenza	0	0	0	2	0	0	0	0			2	1
Bocavirus	3	0	3	1	1	1	0	2			0	0
Enterovirus	0	0	2	0	0	0	0	0			0	0
Haemophilus	0	1	3	1	0	0	0	0			0	0
Metapneumov.	0	0	0	17	0	2	0	0			0	4
Parainfluenzae	1	2	0	3	2	0	0	2			0	0
Rhinovirus	4	3	10	17	4	2	0	3			0	1
Rotavirus	0	0	0	0	0	0	0	1			0	0
SARS-CoV-2	2	1	3	3	0	2	5	1			0	2
Streptococcus	9	0	1	0	0	0	1	0			0	0
Coronav. OC43	0	0	2	0	0	0	0	0			0	0
Total	20	7	25	45	8	7	7	9			2	8

* Not determined.

**Table 3:** Respiratory syncytial virus status and main treatment features in 623 children hospitalized for bronchiolitis, stratified by period of accrual (2021–2022 vs. 2022–2024).

Variable	Period 1 (2021–2022)	Period 2 (2022–2024)	*p*-Value
RSV-positive, *n* (%)	142 (83)	306 (69)	**0.0007**
Oxygen therapy, *n* (%)			
LFNC	118 (67)	162 (37)	**<0.0001**
HFNC	62 (35)	187 (43)	0.0832
Antibiotic therapy, *n* (%)	88 (50)	106 (24)	**<0.0001**
Systemic steroid therapy, *n* (%)	56 (32)	81 (19)	**0.0005**
Inhaled therapy, *n* (%) *	74 (69.8)	53 (16.6)	**<0.0001**
ICU admission, *n* (%)	8 (4)	18 (4)	0.7895

Legend: RSV = respiratory syncytial virus; LFNC = low-flow nasal cannula; HFNC = high-flow nasal cannula; ICU = Intensive Care Unit. * Statistical analysis for inhaled therapy includes data from Centers 1 and 2 only. Bold values indicate statistically significant differences (*p* < 0.05).

**Table 4:** Main features of the treatment applied to 623 children with bronchiolitis in three pediatric centers during two subsequent periods (seasons 2021–2022 and 2022–2024), organized according to the treating center.

	1			2			3		
Total, n	Period 1 62	Period 2 203	***p*-value**	Period 1 44	Period 2 119	***p*-value**	Period 1 71	Period 2 124	***p*-value**
LFNC, n (%)	50 (80.6)	123 (61.5)	**0.0054**	15 (34.1)	26 (21.8)	0.108	53 (74.6)	13 (10.9)	**<0.0001**
HFNC, n (%)	16 (25.8)	36 (18.0)	0.178	10 (22.7)	45 (37.8)	0.07	36 (50.7)	106 (88.3)	**<0.0001**
Steroid therapy, n (%)	10 (16.1)	34 (17.0)	0.872	22 (50.0)	14 (11.7)	**<0.0001**	24 (33.8)	33 (28.4)	0.440
Antibiotic therapy n (%)	29 (46.7)	54 (27.0)	**0.003**	12 (27.2)	26 (21.8)	0.467	47 (66.2)	26 (22.4)	**<0.0001**
Inhaled therapy, n (%)	43 (69.3)	23 (12.2)	**<0.0001**	31 (70.4)	30 (25.2)	<**0.0001**	68 (95.7)	-	
CRP test examination, n (%)	62 (100)	72 (35.5)	**<0.0001** *	34 (77.3)	67 (56.3)	**0.014**	57 (80.3)	-	**n.c.**
Need for intensive care, n (%)	4 (6.4%)	3 (1.5%)	0.054 *	0 (0.0%)	3 (2.5%)	0.386	4 (5.6%)	12 (9.8%)	0.420
Median duration of hospital stay, days (IQR)	6 (5–8)	7 (5–8.5)	0.953	3 (3–5)	3 (2–6)	0.2553	8 (5–9)	6 (4–8)	**0.0002**

Legend: LFNC: low-flow nasal cannula; HFNC: high-flow nasal cannula; IQR: interquartile range. CRP: C-reactive protein; bold indicates *p*-value < 0.05; * the difference has been calculated using Fisher’s exact test. n.c. not calculable.

## Data Availability

The original contributions presented in this study are included in the article. Further inquiries can be directed to the corresponding author.
